# A Nanoparticle Based Sp17 Peptide Vaccine Exposes New Immuno-Dominant and Species Cross-reactive B Cell Epitopes

**DOI:** 10.3390/vaccines3040875

**Published:** 2015-10-29

**Authors:** Sue D. Xiang, Qian Gao, Kirsty L. Wilson, Arne Heyerick, Magdalena Plebanski

**Affiliations:** 1 Department of Immunology and Pathology, Central Clinical School, Faculty of Medicine, Nursing and Health Sciences, Monash University, 89 Commercial Rd, Melbourne, VIC 3004, Australia; E-Mails: qgao10@student.monash.edu (Q.G.); Kirsty.Wilson@monash.edu (K.L.W.); 2 Therapeutics and Regenerative Medicine Division, The Monash Institute of Medical Engineering (MIME), Monash University, Clayton, VIC 3800, Australia; 3 PX Biosolutions Pty Ltd, PO Box 290, South Melbourne, VIC 3205, Australia; E-Mail: Arne.Heyerick@pxbiosolutions.com

**Keywords:** nanoparticles, ovarian, cancer, vaccine, testis-antigen, antibody, cross-reactivity, immunodomunant, Sp17

## Abstract

Sperm protein antigen 17 (Sp17), expressed in primary as well as in metastatic lesions in >83% of patients with ovarian cancer, is a promising ovarian cancer vaccine candidate. Herein we describe the formulation of nanoparticle based vaccines based on human Sp17 (hSp17) sequence derived peptides, and map the immuno-dominant T cell and antibody epitopes induced using such formulations. The primary T and B cell immuno-dominant region within Sp17 was found to be the same when using biocompatible nanoparticle carriers or the conventional “mix-in” pro-inflammatory adjuvant CpG, both mapping to amino acids (aa) 111–142. However, delivery of hSp17_111–142_ as a nanoparticle conjugate promoted a number of new properties, changing the dominant antibody isotype induced from IgG2a to IgG1 and the fine specificity of the B cell epitopes within hSp17_111–142_, from an immuno-dominant region 134–142 aa for CpG, to region 121–138 aa for nanoparticles. Associated with this change in specificity was a substantial increase in antibody cross-reactivity between mouse and human Sp17. These results indicate conjugation of antigen to nanoparticles can have major effects on fine antigen specificity, which surprisingly could be beneficially used to increase the cross-reactivity of antibody responses.

## 1. Introduction

The latest studies on cancer incidence and mortality (International Agency for Research on Cancer; 2014) estimate that there are approximately 239,000 new cases of ovarian cancer (OC) worldwide each year, leading to over 152,000 deaths, mostly from recurrent disease, making it the fifth most common cause of cancer-related death among women [1,2]. The hefty mortality rate is largely due to the non-specificity of the symptoms at early disease stages, leading to late diagnosis and a high chance of recurrence [3,4,5]. Despite advances in combinatorial surgery and chemotherapy regimens over the last decade, patient survival rates have remained unchanged, and >60% of patients die within five years due to tumor recurrence [3,4,5]. Therefore, alternative therapeutic approaches are urgently needed. Immunotherapy treatment strategies including cancer vaccines are considered more specific and less toxic than current treatments [6]. Importantly, induction of solid immunity may be able to prevent the recurrent disease associated with mortality. Although various tumour associated antigens, such as NY-ESO-1, MUC1 and HER-2/neu, have been identified and used in clinical vaccine OC trials with some encouraging results [7,8,9,10,11], these antigens are expressed in <30% of ovarian cancers, limiting the number of patients eligible for treatment [12]. Sperm protein antigen 17 (Sp17) is a cancer testis antigen aberrantly expressed in primary as well as in metastatic lesions in >83% of OC patients, but undetectable by these same methods in normal tissues [13]. Although Sp17 is an autoantigen, it is highly immunogenic *in vivo* [14] and showed great potential to be vaccine and immunotherapy targets for immunotherapy of OC [15,16,17,18]. CpG-adjuvated Sp17 vaccines have been shown to have both therapeutic and prophylactic activity in the C57BL/6-ID8 syngeneic murine model of OC [17].

There is great interest in developing peptide based vaccines to target cancer, given their stability, affordability and ability to personalise treatments (to the specific peptide sequences found in a patient) [19]. Recently, we mapped the protective B and T cell epitope regions within human Sp17 (hSp17) in a murine OC model [20]. We specifically found an immuno-dominant peptide fragment spanning amino acids (aa) 111–142 (hSp17_111–142_), which, when adjuvanted with CpG (ODN 1826), was immunogenic, induced high levels of antibodies and IFN-γ producing T cells (but not IL-17 or IL-4), both in C57BL/6 and in HLA-A2.1 transgenic mice, and significantly prolonged the life-span of the mice bearing the ovarian carcinoma ID8 cell line. hSp17 is composed of 151 amino acids (aa), and it is highly conserved (with 94% homology between murine and human sequences) [21]. We further fine-mapped the immuno-dominant B and T cell epitope regions within hSp17_111–142_ and identified a single immuno-dominant B cell (134–142 aa) epitope and two T helper 1 (Th1) cell epitopes (111–124 aa and 124–138 aa) [20]. These result together support further exploration of hSp17_111–142_ peptide formulations as vaccines against ovarian cancer.

Synthetic oligodeoxynucleotides containing unmethylated CpG motifs have been extensively studied as adjuvants [22,23]. The CpG is a pathogen associated molecular pattern (PAMP), recognized by the pattern recognition receptor toll-like receptor 9 (TLR9). TLR9 is expressed on human B cells and plasmacytoid dendritic cells (pDCs), but not human conventional DC (cDC), in contrast to mice where it is more broadly expressed, suggesting different adjuvanting mechanisms across species [24,25,26]. Although CpG can improve the activity of vaccines [23], CpG adjuvanted vaccine formulations are often associated with florid inflammatory responses, including the production of cytokines TNF and IL-6 [27,28]. In OC, such high inflammatory responses, and particularly high levels of IL-6, are associated with disease progression [29,30,31,32]. It would hence be of interest to be able to induce high levels of immunity, but not inflammation, in the context of OC vaccines. In previous studies with mice and sheep antigens covalently conjugated to non-inflammatory biocompatible nanoparticles 40–50 nm made of a polystyrene core (PSNPs) were shown to induce potent CD8+ and CD4+ T cells responses as well as antibody responses *in vivo* [33,34,35,36,37]. With such vaccine formulations, some experimental tumours in mice could be eliminated after just one injection [34]. Surprisingly, and in contrast to conventional adjuvants such as Alum, these nanoparticle-based vaccine formulations (nanovaccines) have potent immunological activity even in the absence of a concomitant conventional inflammatory responses, and do not require the addition of TLRs or DC cell targeting ligands to be potent immunogens [38,39,40]. Furthermore, long-lasting immune responses were also observed following immunisation with PSNPs adjuvanted vaccine formulations [33]. A potential confounder in the development of nanoparticle based vaccines using peptide fragments is that their attachment to the nanoparticles may result in different epitopes being exposed, which may affect how the antigen is processed *in vivo*, leading to a potential loss of immunogenicity, or conversely, enabling sub-immunogenic regions to be effectively presented, changing the spectrum of immuno-dominance. To further explore the therapeutic potential of hSp17 peptide fragments and support the development of non-inflammatory nano-therapeutic vaccines against OC, we studied the ability of six overlapping peptide fragments covering the hSp17 protein sequence to induce immunity after being delivered covalently bound to PSNPs. We further studied immuno-dominance and cross-species reactivity for these particle bound formulations, in the context of the previously reported responses which had been formulated in CpG. The results suggest subtle but important differences between nanoparticle bound and CpG “mixed in” formulations, with new insights into the optimisation of formulation design for the treatment of OC.

## 2. Material and Methods

### 2.1. Peptides and Recombinant Sp17 Protein

**Figure 1:**
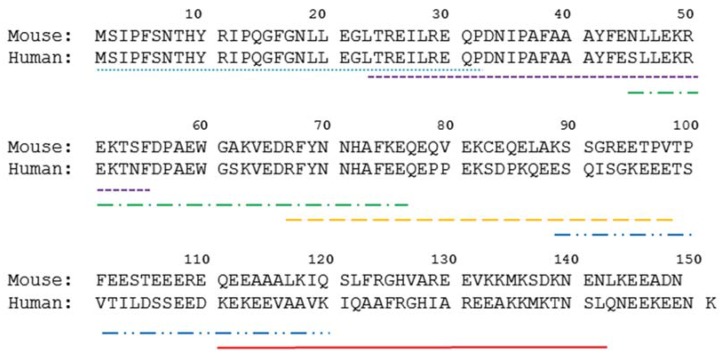
Diagram of both human and mouse Sp17 amino acid sequences (adapted from UniProtKB/Swiss-Prot, for human: http://www.uniprot.org/uniprot/Q15506; for mouse: http://www.uniprot.org/uniprot/Q62252). Six of 32 mer long peptides from the hSp17 sequence (hSp17_1–32_, hSp17_23–54_, hSp17_45–76_, hSp17_67–98_, hSp17_89–120_ and hSp17_111–142_) were designed and synthesised as vaccine targets.

Six 32 mer long human Sp17 peptides (hSp17_1–32_, hSp17_23–54_, hSp17_45–76_, hSp17_67–98_, hSp17_89–120_ and hSp17_111–142_) were designed (Figure 1), and synthesized by GenScript (Piscataway, NJ, USA) and Auspep (Tullamarine, Australia). Overlapping peptide fragments in the C-termini region of human Sp17 (hSp17_111–142_) and mouse Sp17 (mSp17_109–143_) were also designed and synthesized by Mimotopes (Clayton, VIC, Australia) and Synpeptide (Shanghai, China) (Figure 1, semi-dotted lines). Mouse recombinant Sp17 protein (rmSp17) was kindly produced by Dr. Chiriva-Internati (Texas Tech University, Lobbock, TX, USA).

### 2.2. Vaccine Formulations

Six overlapping peptides from the human Sp17 protein sequence, namely hSp17_1–32_, hSp17_23–54_, hSp17C_45–76_, hSp17_67–98_, hSp17_89–120_ and hSp17_111–142_, were used as vaccine antigens. Each of the individual peptides were either mixed with CpG (ODN 1826, InvivoGen, San Diego, CA, USA) directly or conjugated to 40–50 nm carboxylated polystyrene nanoparticles (PSNPs, Polysciences Inc., Warrington, PA, USA). Peptide conjugations were optimised for each peptide in order to achieve the best conjugation efficiency and size. In brief, following the conjugation procedures described previously [38], PSNPs at a final of 1% solids were pre-activated in a mixture containing 2-*N*-Morpholino-ethanesulfonic acid (MES) (50 mM final, pH = 6), 1-ethyl-3-(3-dimethylaminopropryl) carbodiimide hydrochloride (EDC) (4 mg/mL final) (Sigma-Aldrich, St. Louis, USA), *N*-hydrosulfosuccinimide (Sulfo-NHS) (50 mM final) (Pierce™, Thermo Fisher Scientific, Waltham, Massachusetts, USA), adjusted pH to be 5.5–6. After pre-activation, the excess activation agents (EDC and Sulfo-NHS) were removed from the pre-activation mix using a gel filtration column (Zeba spin desalting column following manufacturer’s instruction, Thermo Fisher Scientific), and buffer exchanged at the same time via the column (buffer optimised for each antigen) before adding the peptide antigen for a further 2 h. The final conjugation mix was then dialysed against PBS in 1 kDa dialysis membrane. Final conjugation efficiency was determined by BCA™ protein assay (Pierce™ Micro BCA protein assay, Thermo Fisher Scientific) and sizes were measured by Zetasizer (Malvern Instruments Ltd, Worcestershire, UK). Each vaccine dose (~100 μL) contained 50–100 μg peptides with 20 μg CpG or ~1% solid of PSNPs in PBS. The amounts of peptide antigen injected were matched for both formulations by adjusting the injection volume for each experiment.

### 2.3. Immunisations, Immunogenicity and Immune-Therapy

The vaccine study was approved by the AMREP animal ethics committee, Melbourne, Australia. Treatment and care of the animals were in accordance with Institutional Guidelines and the Animal Welfare Assurance Act. To study the immunogenicity of the hSp17 peptide antigens, 6–8 week old female C57BL/6 (H-2K^b^) and HLA-A2.1 (A2KbC57BL/6JTgN(A2KbH2b)6Hsd)) transgenic (Tg) mice sourced from Monash Animal Services (Clayton, VIC, Australia) and Animal Resources Centre ( Murdoch, WA, Australia) were used. Mice were immunised with 100 μL of each vaccine formulation at various concentrations intradermally at the base of the tail. Details of each immunisation schedule are listed in the figure legends. Injections with PBS alone served as a negative control (Naïve). For multiple immunisations, mice were usually boosted with the same formulation 7–10 days apart (see figure legends for details of each experiment). 7–14 days following the last immunosation, mice were euthanized by CO_2_ asphyxiation and spleen removed and splenocytes were harvested for immunogenicity assays (ELISPOT). Sera were also collected before immunisation and at the end point for detecting antigen specific antibodies by ELISA.

### 2.4. ELISPOT and ELISA Assays

Antigen specific CD4, CD8 or Th17 T cell responses were evaluated by IL-4, IFN-γ and IL-17 ELISPOT assays [36]. Briefly, 96-well filtration plates (MAHA, MSIP or MAIP plates, Millipore, Billerica, MA, USA) were coated with 100 μL/well of either anti-mouse IFN-γ (AN18, 5 μg/mL, MABTech, Stockholm, Sweden), anti-mouse IL-17 (5 μg/mL, MABTech) or rat anti-mouse IL-4 (5 μg/mL, BD Pharmingen, San Diego, CA, USA). Following overnight incubation at 4 °C, the wells were washed and blocked with RPMI 1640 completed medium (CM) supplemented with 10% heat inactivated fetal calf serum (FCS), 2 mM glutamine, 100 μg/mL streptomycin, 100 units/mL penicillin, 0.2 mM β-mercaptoethanol and 20 mM Hepes (all from Gibco^®^, Thermo Fisher Scientific). Splenocytes (50 μL) from immunised mice (2 × 10^7^ cells/mL, either individual or pooled) were added to triplicate wells and incubated with 50 μL of recall antigens (rmSp17 protein or Sp17 peptides) at various concentrations (2.5–25 μg/mL final for all potential CD8 epitopes and 25–100 μg/mL final for long peptides and protein) at 37 °C incubator filled with 5% CO_2_ for a minimum of 16 h for IFN-γ plates, 24 h for IL-4 plates and 40 h for IL-17 plates. Concanavalin A (Con-A) (1 μg/mL final, Amersham Biosciences, Uppsala, Sweden) was used as a positive control and background wells were added with CM. The plates were then washed 6 times in PBS and incubated with 100 μL biotinylated detection antibodies (anti-mouse IFN-γ biotinylated mAb R4-6A2 (Mabtech); anti-mouse IL-17 biotin (Mabtech); rat anti-mouse IL-4 biotin (BD), all at 1 μg/mL final) at room temperature for 1–2 h. After washing as above, steptavidin-alkaline phosphatase (to detect IFN-γ and IL-17, MabTech) or Extravidin-ALP (for detection of IL-4, Sigma-Aldrich, St. Louis, MO, USA) was added (final at 1 μg/mL) and incubated for another 1.5 hours at room temperature. Plates were then washed again, with a final wash using Reverse Osmosis (RO) water to remove residual PBS. The spots were developed using a colorimetric AP kit (Bio-Rad, Philadelphia, PA, USA) following the manufacturers’ instructions. Spot counting was performed using an AID ELISPOT Reader System (AID GmbH, Strassberg, Germany). The magnitude of the specific cytokine induction in response to the recall antigen were compared either directly for its spot forming unit (SFU) or normalised against the corresponding naïve response to the same recall antigen, calculated as stimulation index (SI) of SFU over naïve (SI = (SFU from the treatment mice)/(SFU from the naïve mice) for each corresponding recall antigens). An antigen-specific response was considered to be positive only when the SI ≥ 2 and net SFU ≥ 20 per million cells.

Sera from vaccinated animals were collected before immunisation and at the end point, and assayed for antigen-specific antibody production by ELISA. Briefly, 96-well plates (Maxisorp™, NUNC, Roskilde, Denmark) were coated with rmSp17 protein or peptides diluted in carbonate/bicarbonate coating buffer (5 μg/mL, 50 μL/well) and incubated overnight at 4 °C. After washing with PBS/0.05% Tween-20 and blocking with 5% skim milk in PBS, serial dilutions of mouse sera were added and incubated at 37 °C for 2 h or 4 °C overnight. After washing as above, HRP-conjugated sheep anti-mouse IgG (Amersham) was added and allowed to incubate at 37 °C for another 1 h. The reaction was developed using TMB substrate (Invitrogen™, Thermo Fisher Scientific) and stopped with 1 M HCl, before reading the absorbance at 450 nm (OD_450 nm_). The magnitude of the antibody levels were compared either directly for its OD_450 nm_ reading or normalised against the corresponding naïve serum at the same dilution point (also calculated as SI = (OD_450 nm_ for test serum)/(OD_450 nm_ for naïve serum) at the same dilution point). Antibody endpoint titres represent the degree to which the serum could be diluted and still contain detectable amounts of antibody, and were calculated as the serum dilution at which the OD_450 nm_ was equal to the mean OD of the serum of naïve mice + 3 standard deviations (SD).

Competition ELISA were performed by incubating the test serum (usually at 1/100–1/400 dilutions depending on the antibody titres) with competing peptides (serial diluted from 10 μg/well) at 1:1 ratio (volume:volume) in a 96-well tissue culture plate for 1 h in a 37 °C incubator. After the incubation, 50 μL of the “competition mix” from each well were transferred to corresponding ELISA plates which were pre-coated (and blocked) with the same competing peptide (see above). The standard ELISA protocol as described above was continued from this step. The change of OD_450 nm_ reading reflects the cross-reactivities between the antibody and competing peptides.

### 2.5. Statistical Analysis

Statistical analysis was done by one-way or 2-way ANOVA using Graph Pad Prism v6.04 software (GraphPad Software, Inc., La Jolla, CA, USA) and Excel (Microsoft Corporation, Redmond, Washington, USA). Differences were considered statistically significant at *p* < 0.05. Group sizes are indicated in the Figure legends. All values are expressed as mean ± SD.

## 3. Results and Discussion

### 3.1. PSNPs-hSp17 Peptides Formulations

We have recently shown that specific hSp17 peptides may induce IFN-γ and antibody responses when adjuvanted with CpG [20], indicating their potential to progress as a peptide based vaccine against OC. Although CpG is a potent adjuvant, can induce strong systematic inflammatory responses, it is not best suited to progress for human use, particularly in OC, where inflammation is associated with disease progression [29,30,31,32]. In this study, we investigated a model of non-inflammatory nanoparticle based vaccine delivery system for its ability to induce Sp17 specific immune responses. Six overlapping hSp17 peptides were conjugated to PSNPs (hSp17_1–32_-PSNPs, hSp17_23–54_-PSNPs, hSp17C_45–76_-PSNPs, hSp17_67–98_-PSNPs, hSp17_89–120_-PSNPs and hSp17_111–142_-PSNPs) as per Table 1 to 40–50 nm carboxylated polystyrene nanoparticles to form the nanoparticle based vaccine formulations (hSp17peptides-PSNPs). Conjugations were optimised for each peptide in order to achieve the best conjugation efficiency and size (40–60 nm). Two conjugation buffer systems (MES and PBS) and a range of pH conditions (pH 5.5 to pH 8) were tested for each peptide conjugated to PSNPs. All six hSp17 peptides were able to be conjugated to the PSNPs in the optimal size range and efficiency (% peptide successfully conjugated to the PSNPs). The optimised conjugation conditions for each peptide are summarised in Table 1, including the conjugation efficiency and the final particle sizes for the conjugated formulations. During the conjugation process, ultra-high conjugation efficacy could be achieved under some of the pH conditions; however these were associated with much larger formulation sizes, possibly due to the aggregation during the conjugation process. Given our previous studies have shown that the size of nanoparticle formulation was also crucial for the induction of a desired immune response [33,34,35,38]; we selected the appropriate conjugation conditions based on both the optimal size and appropriate conjugation efficiency.

**Table 1:** Optimised conjugation conditions, efficiency and size of the hSp17 peptides-PSNPs formulations.

Conjugation conditions	hSp17_1–32_-PSNPs	hSp17_23–54_-PSNPs	hSp17_45–76_-PSNPs	hSp17_67–98_-PSNPs	hSp17_89–120_-PSNPs	hSp17_111–142_-PSNPs
Conjugation buffer *	MES	MES	PBS	PBS	PBS	MES
Conjugation pH ^#^	5.5	5.5	7.5	6.5	6	6.2
Conjugation efficiency ^§^ (%)	94.5 ± 2.2	87.9 ± 2.9	95.2 ± 1.2	77.1 ± 3.7	69.8 ± 3.6	95.8 ± 1.1
Size (nm)	72.3 ± 4.8	52.5 ± 0.2	49.1 ± 1.4	48.8 ± 0.8	45.9 ± 0.5	55.9 ± 0.1

*^,^^#^: Conjugation buffer and pH referred to the buffer and pH conditions used for equilibration of column during the desalting process and incubation during the peptide and PSNPs mixture. ^§^: Conjugation efficiency was determined as the percentage of peptide antigen successfully conjugated to PSNPs.

### 3.2. Immune Responses Induced by hSp17peptides-PSNPs Vaccine Formulations

**Figure 2:**
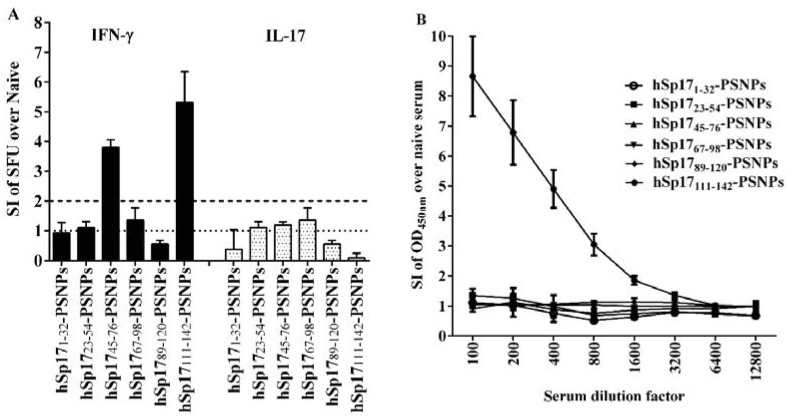
Immunogenicity of the hSp17 peptides based vaccines adjuvanted/carried by PSNPs. HLA-A2/K^b^ mice (*n* = 3–4/group) were immunised twice (intradermally at the base of tail, two weeks apart) with each hSp17 peptide (hSp17_1–32_, hSp17_23–54_, hSp17_45–76_, hSp17_67–98_, hSp17_89–120_ and hSp17_111–142_) conjugated to PSNPs (each injection dosage contained ~50 μg peptide and 1% PSNPs). 10–14 days after the last immunisation, sera were collected and assayed for antigen specific antibodies by ELISA. Splenocytes IFN-γ responses to each of the peptide antigens were measured in triplicate by ELISPOT assays. Figures are summarised from seven different experiments, all data were normalised against responses from the naïve samples. (**A**): IFN-γ and IL-17 T cell responses. Data presented as average SI of SFU ± SD (SI = SFU of the peptide response in vaccinated mice/SFU for the same peptide response in naive mice). Dotted line indicated the background level (SI = 1), and semi-dotted line indicated the minimal level of a positive response (SI ≥ 2); (**B**): Antigen specific antibody production by PSNPs adjuvanted the hSp17 peptide vaccines. Data presented as average SI of OD_450 nm_ ± SD (SI = the OD_450 nm_ of vaccinated serum/OD_450 nm_ of naïve serum at the same dilutions, *n* = 3–4 individual mice).

Each of the six hSp17peptides-PSNPs formulations in Table 1 were immunised into HLA-A2.1 Tg mice, and antigen specific IFN-γ and IL-17 responses, as well as antibody levels were measured. As shown in Figure 2A, similar to the results using CpG as the adjuvant [20], the most immunogenic hSp17 peptide PSNPs based vaccine formulation was the one based on peptide hSp17_111–142_, as determined by its ability to induce IFN-γ responses; followed by peptide hSp17_45–76_, and no significant immune responses induced to any of the other fragments. No detectable IL-17 T cell responses were induced by any of the peptide regions, including the hSp17_111–142_. Antibody responses were clearly only detected after immunisation with the formulation hSp17_111–142_-PSNPs, with none of the other five hSp17 peptides-PSNPs formulations inducing any antigen specific antibody responses (Figure 2B). These results confirmed across two widely different adjuvant systems, hSp17_111–142_ peptide contained the primary immunogenic region within hSp17.

Similarly to our previous published data on the CpG adjuvanted Sp17 peptide vaccine formulations [20], we also tested for the potential induction of HLA-A2.1 restricted CD8 T cells responses using minimal predicted HLA-A2.1 binding peptides in Sp17 sequence as recall antigens in ELISPOT in the same immunized animals by the nanoparticle adjuvanted formulations, albeit we did not find evidence of CD8 T cell induction using this approach (see Appendix Table A1 and Table A2), it is still possible that CD8 T cell responses to epitopes that do not utilize classical MHC class I anchors, or have low affinity to these MHC would have been missed in any such analysis. Furthermore, lack of HLA-A2.1 restricted responses does not exclude the possibility that Sp17 may contain T cell epitopes restricted by other human MHC class I alleles. For example, Chiriva-Internati and colleagues had previously identified a cytotoxic HLA-A1 restricted T cell response against Sp17 in 3 patients with ovarian cancer [41,42]. Fully mapping such epitopes requires a fully empirical approach, as prediction algorithms are as yet unavailable. Although beyond the scope of the present study, such approaches would be a valuable extension to the study of Sp17 in future.

### 3.3. The Immunogenicity of hSp17_111–142_ Peptide may be Hampered by the Conjugation Process to PSNPs

**Figure 3:**
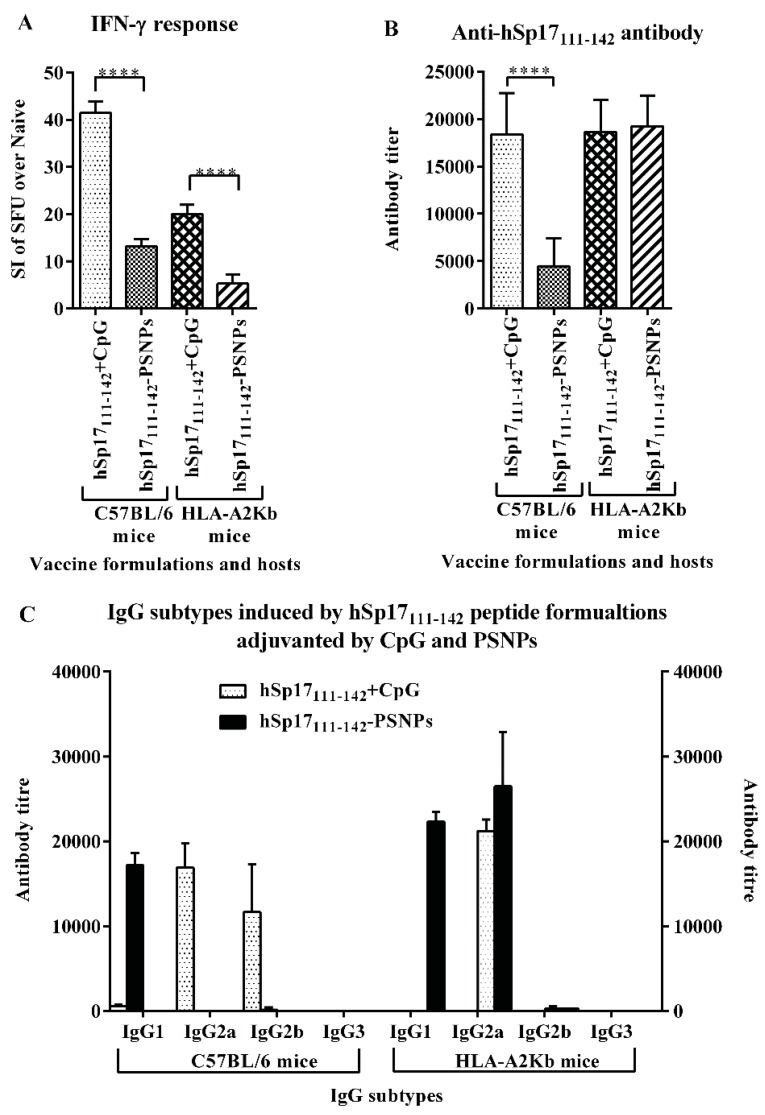
Immunogenicity of the hSp17_111–142_ adjuvanted by CpG or PSNPs in different mice strains. C57BL/6 mice (*n* = 4/group) and HLA-A2K^b^ mice (*n* = 4/group) were immunised 4 times (intradermally at the base of tail, 10 days apart) with hSp17F_111–142_ peptide (56 μg/mouse), adjuvanted with either CpG (20 μg/mouse) or PSNPs (1% solid). 8 days after last immunisation, splenocytes IFN-γ responses to the peptide antigen were measured by ELISPOT assays. Sera were collected and assayed for antigen specific antibodies by ELISA. (**A**): IFN-γ responses. Data presented as average SI ± SD (SI = SFU of the peptide response in vaccinated mice/SFU for the same peptide response in naive mice). (**B**): Anti-hSp17_111–142_ specific antibody (IgG) production. **** *p* < 0.0001. (**C**): IgG subtypes in C57BL/6 and HLA-A2Kb mice. Data presented as antibody titres ± SD (*n* = 4 individual mice).

To further compare directly the immunogenicity of the hSp17_111–142_-PSNPs peptide formulation with CpG adjuvanted formulation; these two formulations were injected side-by-side into C57BL/6 and HLA-A2.1 Tg mice strains. Figure 3 shows the overall magnitude of the IFN-γ response induced by the hSp17_111–142_ peptide was significantly lower in the PSNPs adjuvanted formulation than in the CpG adjuvanted formulation in both mice strains (Figure 3A), and the response was also significantly higher in C56BL/6 mice than in HLA-A2.1 mice for the same formulation (Figure 3A). On the contrary, for antibody responses, both formulations induced similar levels of IgG in HLA-A2.1 mice, which were also comparable to the CpG adjuvanted formulation in C57BL/6 mice, but to a large extent, the PSNPs adjuvanted formulation in C57BL/6 mice induced much lower antibody responses (Figure 3B). The use of PSNPs based delivery for the hSp17_111–142_ peptide also resulted in a different antibody IgG subtype being induced compared to the CpG adjuvated formulation. As shown in Figure 3C (left), the CpG adjuvanted formulation predominantly induced IgG2a and IgG2b, whereas the PSNPs-conjugated formulation induced IgG1 in C57BL/6 mice. The pattern was slightly different in HLA-A2.1 mice, with the CpG adjuvanted formulation inducing IgG2a, and the PSNPs-conjugated formulation inducing both IgG2a and IgG1 subtypes (Figure 3C, right). A predominantly IgG2a response is consistent with previous use of the CpG adjuvant in the literature [43]. IgG1 is often associated with a Th2 profile and other subclasses are mainly associated with a Th1 (IgG2a and IgG3) profile [44]. The induction of IgG1 and IgG2a antibodies in PSNPs adjuvanted hSp17_111–142_ formulation indicated a mixed Th1/Th2 responses induced by this formulation. The differences in magnitude of immune responses between C57/B6 and HLA-A2.1 mice across the formulations were surprising, given HLA-A2.1 mice are transgenic on a C57BL/6 background, and our previous studies found no evidence of CD8 T cell reactivity to conventional predicted CD8 T cell epitopes in either strain measured by the production of IFN-γ in ELISPOT assay (Appendix Table A1), suggesting the observed IFN-γ responses to the hSp17_111–142_ peptide following immunisation were mediated by CD4 T cells since the response was disappeared when the CD4 T cells were pre-depleted from the splenocytes used in the ELISPOT assays [45]. It is still however possible that low affinity MHC class I binding peptides using non-conventional anchor motifs may contribute to the observed IFN-γ T cell responses, and these are processed and presented differently across these two strains. It is more difficult to explain strain differences in antibody reactivity, as these would have had to arise indirectly due to differences in MHC class I alone, but could be speculated to derive from differences in the T and B cell repertoire across the two mice strains. Importantly, the relatively lower T cell responses induced by the PSNPs conjugated peptide formulation *vs*. CpG across both mouse strains (at the same peptide dosage) indicate the possibility of structural changes to the immunodominant regions within the peptide, which may have occurred as a consequence of the peptide conjugation process.

### 3.4. Strong Antibody Cross-reactivity to rmSp17 Protein

**Figure 4:**
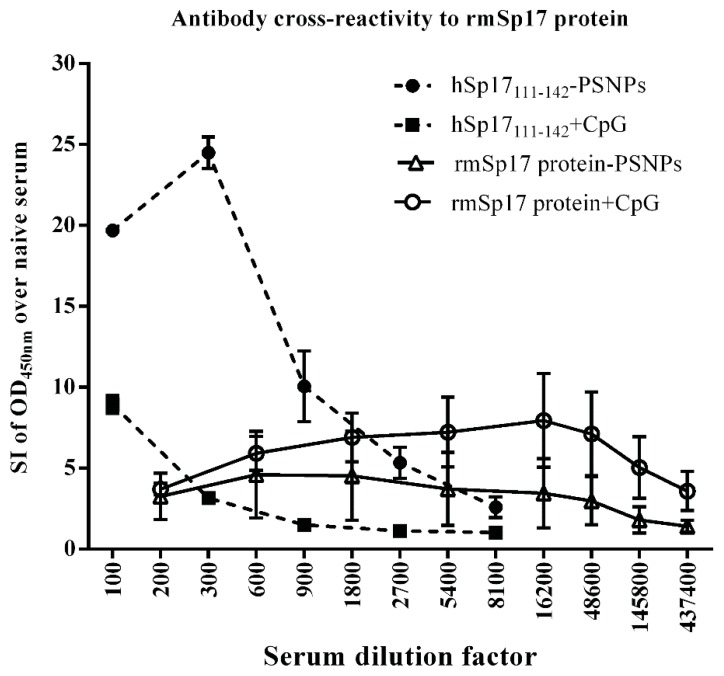
Antibody cross-reactivity to rmSp17. Serum from C57BL/6 mice (*n* = 3/group) immunised with hSp17_111–142_ (100 μg/dosage) and rmSp17 protein (100 μg/dosage) both adjuvated with either CpG (25 μg/dosage) or PSNPs (1% solid/dosage) were assayed in ELISA coated with rmSp17 protein (5 μg/mL) to test the cross-reactivity of these sera to the rmSp17 protein. Standard 2 immunisations intradermally at the base of tail. Data presented as average SI of OD_450 nm_ over naive ± SD.

Structural/conformational changes in hSp17_111–142_ as a result of conjugation to PSNPs were further supported by the observation that sera from hSp17_111–142_-PSNPs immunised C57BL/6 mice showed strong cross reactivity to the recombinant murine (rm) Sp17 protein in ELISA (Figure 4); contrasting the weak cross-reactivity shown by antibodies induced by the CpG adjuvanted hSp17_111–142_ formulation. These results suggest that in the case of antibody induction, conjugation to the nanoparticles may have exposed a useful conserved epitope region within Sp17_111–142_ and promoted its immunogenncity.

Fragment 111–142 is one of the least homologous regions between murine and human Sp17, with an at face value homology of 6.25%. However, the divergence in sequence is largely mediated by a shift in alignment mediated by the insertion of amino acids. When the sequences are re-aligned so sequence 112–138 (human) aligns with 109–136 (murine), homology increases to 74.1%, with a conserved stretch of 12 identical amino acids from positions 125–128 (12 aa/14 aa identical, and one amino acid conservative changes, ~85.7%–92.8% homology). It is likely that cross-reactivity is targeted to such conserved stretches upon alignment. The ability to induce cross-reactive antibodies between murine and human Sp17 is useful for many clinical applications, such as xenogeneic immunizations, and may help to break self-tolerance, an aspect that may be worth exploring in depth in future studies.

The basic principle of being able to enhance cross-reactivity between any two antigens is of broad interest beyond the potential specific application to Sp17, for example, to tackle highly polymorphic antigens, or antigens that mutate rapidly in the face of selection pressure.

### 3.5. Changing the Dynamic of Epitope Recognitions after the Conjugation Process to PSNPs

**Figure 5:**
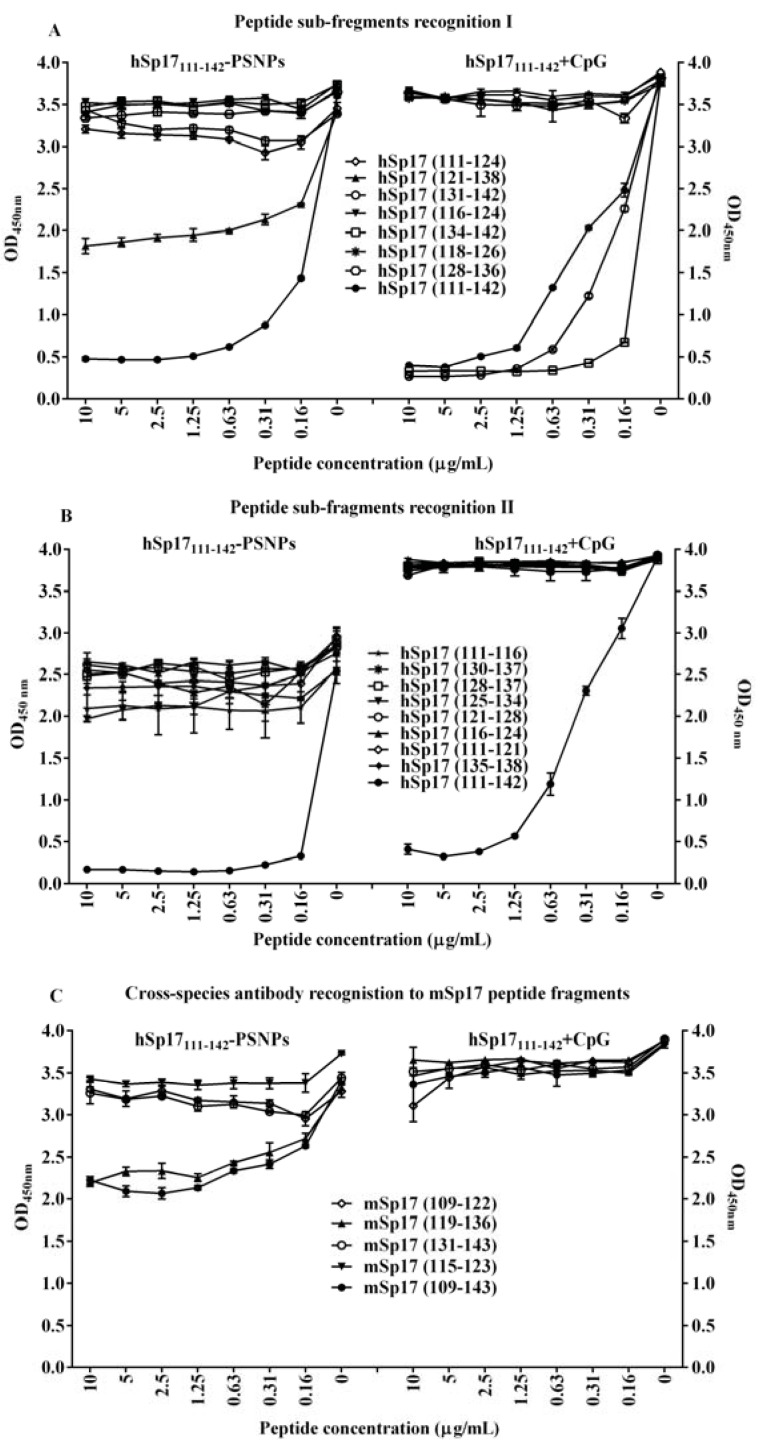
B cell epitope recognitions effected by the different adjuvant system. C57BL/6 mice (*n* = 4 mice/group) were immunised 4 times (weekly apart) with hSp17_111–142_-PSNPs vaccine formulation (containing 36 μg /ml of hSp17_111–142_ peptide and 1% PSNPs, 100 μL/mouse/injection) or with CpG adjuvated hSp17_111–142_ vaccine formulation (at equivalent peptide dose, *i.e.*, (36 μg peptide + 20 μg CpG)/mouse/injection). 13 days after the last immunisation, sera were collected, and pooled for each group. A&B: hSp17_111–142_ epitope recognitions. Seven (**A**) and nine (**B**) peptide fragments within the hSp17_111–142_ region as well as hSp17_111–142_ itself were used to compete for the antibody reactivities in sera produced by both vaccine formulations. (**C**): Antibody cross-species reactivity to mSp17 fragments. Five mSp17 peptides were used to compete for antibody reactivity in sera produced by both vaccine formulations. Data presented as Average OD_450 nm_ ± SD (triplicated in assay).

Since the results above suggested that the peptide conjugation process may have exposed new useful B cell epitopes within hSp17_111–142_, in addition to the immunodominant B cell epitope we identified after CpG immunisation (aa 134–142) [20], we proceeded to map the B cell epitopes within hSp17_111–142_ using a panel of small peptide fragments within the hSp17_111–142_ peptide region (Table 2). These were used to block the recognition by the anti-hSp17_111–142_ antibody induced by the hSp17_111–142_-PSNPs formulation in a competition ELISA. Surprisingly, only peptide fragment 121–138 aa and itself (111–142 aa) competed strongly (Figure 5A left panel), with weak competition observed by peptide fragments 111–124 aa and 131–142 aa as well as 111–116 aa and 125–134 aa (Figure 5B left panel); in contrast to the antibodies produced by immunisation with hSp17_111–142_ + CpG, which were strongly competed by 131–142 aa; 134–142 aa; and itself (111–142 aa) as per our previous studies (Figure 5A right panel) [20]. Similarly, further additional peptide fragments assayed with the antibodies produced by “hSp17_111–142_ + CpG” formulation (Figure 5B, right panel) didn’t show any binding to the antibody, further narrowing down the identified B cell epitope to the sequence region of 139–142 aa (TNSL). Since “TNSL” is the C-terminal end of the hSp17_111–142_ peptide sequence, it is most likely that conjugation of the peptide to PSNPs might destroy the epitope or change its accessibility; therefore, the B cell epitope initially identified by the CpG formulation was no longer recognized by the antibody produced by the PSNPs adjuvanted formulation. The change in immunodominance by 121–138 aa, in turn, may have occurred by promoting exposure of a region otherwise masked by the rest of the peptide.

**Table 2:** Sp17 peptide sub-fragments and aligned sequences.

Amino Acid Position	Aligned Sequences
hSp17	
111–142	KEKEEVAAVKIQAAFRGHIAREEAKKMKTNSL
111–116	KEKEEV
111–124	KEKEEVAAVKIQAA
113–121	KEEVAAVKI
116–124	VAAVKIQAA
118–126	AVKIQAAFR
121–138	IQAAFRGHIAREEAKKMK
121–128	IQAAFRGH
125–134	FRGHIAREEA
128–136	IAREEAKK
128–137	HIAREEAKKM
130–137	AREEAKKM
131–142	REEAKKMKTNSL
134–142	AKKMKTNSL
135–138	KKMK
mSp17	
109–143	REQEEAAALKIQSLFRGHVAREEVKKMKSDKNENL
109–122	REQEEAAALKIQSL
115–123	AALKIQSLF
119–136	IQSLFRGHVAREEVKKMK
131–143	EVKKMKSDKNENL

Peptide fragments 111–124 aa and 121–138 aa have also been shown previously to induce strong IFN-γ responses in mice immunised with CpG adjuvanted hSp17_111–142_ formulation [20], suggesting these regions may be useful for the development of antibody vaccines that can also self-promote the T cell help required for long term immunity.

Importantly, this change in specificity of the region recognized by antibodies induced by the PSNPs adjuvanted formulation, also greatly enhanced the cross-reactivity of such antibodies to murine Sp17 peptide (mSp17) fragments corresponding to the equivalent sequence regions at the C-terminal (see Table 2). As shown in Figure 5C, the anti-hSp17_111–142_ antibody activities (generated by hSp17_111–142_-PSNPs formulation) were dramatically reduced by competition for binding with mSp17 peptide fragment 109–143 aa (corresponding to hSp17 (111–142)) as well as the mSp17 fragment 119–136 aa (the equivalent for hSp17 (121–138)) (Figure 5C, left). Notably, none of the murine equivalent peptides could block the binding to hSp17_111–142_ of antibodies induced by hSp17_111–142_+CpG (Figure 5C right). Similar results from the serum obtained in HLA-A2.1 mice [45]. Changing the immuno-dominant region being recognised within hSp17_111–142_, by conjugating to PSNPs, also promoted an immune response that was able to promote cross-reactive responses across specie.

## 4. Conclusions

Together these results show that formulating immunogenic peptides into nanoparticle based vaccines can be used to change the fine specificity, nature (antibody isotype) and cross-reactivity of the antibody response generated, compared to some standard pro-inflammatory mixed in adjuvants, such as CpG. Specifically, we show that whereas it is possible to generate high antibody responses to an immunogenic fragment of hSp17 using non-inflammatory PSNPs as a vaccine delivery system, the use of such nanoparticles promotes a qualitatively different response to the use of CpG, particularly in the nature of the antibody response elicited. We propose that changing the immunodominance and hence patterns of cross-reactivity may be a useful feature for vaccines that aim to be broadly cross-reactive, or where conventional mix-in inflammatory adjuvants have not been able to promote responses to potentially useful subdominant specificities.

## Appendix

**Table A1:** Screening for potential CD8 T cell epitopes* in rmSp17 protein conjugated to PSNPs in both C57BL/6 and HLA-A2.1 mice^#^.

Position	Sequence	Sequence Homology between Human and Mouse	rmSp17 Reactivity
2–10	SIPFSNTHY	100%	−
11–19	RIPQGFGNL	100%	−
12–19	IPQGFGNL	100%	−
12–20	IPQGFGNLL	100%	−
18–26	NLLEGLTRE	100%	−
19–27	LLEGLTREI	100%	−
22–30	GLTREILRE	100%	−
27–35	ILREQPDNI	100%	−
30–38	EQPDNIPAF	100%	−
34–42	NIPAFAAAY	100%	−
38–46	FAAAYFESL	89%	−
39–46	AAAYFENL	89%	−
39–47	AAAYFESLL	89%	−
40–47	AAYFENLL	89%	−
45–53	SLLEKREKT	89%	−
66–74	DRFYNNHAF	100%	−
91–99	QISGKEEET	11%	−
111–119	KEKEEVAAV	56% ^§^	−
115–123	AALKIQSLF	56% ^§^	−
116–124	VAAVKIQAA	56% ^§^	−
134–142	AKKMKTNSL	44% ^§^	−
136–143	KSDKNENL	22%	−
136–144	KSDKNENLK	22%	−
rmSp17	recombinant murine Sp17 protein		++++ ^ξ^

*: 23 of predicted 8–9 mer CD8 epitopes, which have high binding affinity to the HLA-A2.1 and H-2K^b^ and H-2D^b^ molecules were tested here [20]; ^#^: To screen potential CD8 T cell epitopes in rmSp17, both C57BL/6 mice and HLA-A2.1 transgenic mice (*n* = 3–4/group) were immunised twice with rmSp17 protein (50 μg/mouse) conjugated to PSNPs vaccine formulations intradermally, 2 weeks apart. 10–14 days after last immunisation, splenocytes were tested for reactivity in triplicate to each of the 23 predicted CD8 T cell epitopes in IFN-γ ELISPOT assays. Table was summarised from 4 independent experiments. A positive response was determined by the stimulation index (SI) of the SFU of recall peptide in vaccinated mouse/SFU for the same peptide in naïve mouse. The SI ≥ 2 considered as a positive response; ^§^: the homology was adjusted between mouse and human because of the identical amino acid shift in those regions; ^ξ^: indicated strong positive responses with SI > 10.

**Table A2:** Screening for potential HLA-A2.1 restricted CD8 T cell epitopes in hSp17 peptides conjugated to PSNPs in HLA-A2.1 mice*.

Position	Sequence	Immunogen					
		hSp17_1–32_	hSp17_23–54_	hSp17_45–76_	hSp17_67–98_	hSp17_89–120_	hSp17_111–142_
11–19	RIPQGFGNL	**-**					
12–20	IPQGFGNLL	**-**					
18–26	NLLEGLTRE	**-**					
19–27	LLEGLTREI	**-**					
22–30	GLTREILRE	**-**	**-**				
27–35	ILREQPDNI		**-**				
45–53	SLLEKREKT			**-**			
91–99	QISGKEEET				**-**		
111–119	KEKEEVAAV					**-**	**-**
116–124	VAAVKIQAA					**-**	**-**
134–142	AKKMKTNSL						**-**

*: To screen potential HLA-A2.1 restricted CD8 T cell epitopes in the human Sp17 overlapping peptides, HLA-A2.1 transgenic mice (*n* = 3–4/group) were immunised twice with PSNPs adjuvanted hSp17 peptides (hSp17_1–32_, hSp17_23–54_, hSp17_45–76_, hSp17_67–98_, hSp17_89–120_ and hSp17_111–142_, 50 μg/mouse) vaccine formulations intradermally, 2 weeks apart. 10–14 days after last immunisation, splenocytes were tested in triplicates for reactivity to each of the 11 predicted HLA-A2.1 restricted CD8 T cell epitopes in IFN-γ ELISPOT assays. Table was summarised from 6 independent experiments. A positive response was determined by the stimulation index (SI) of the SFU of recall peptide in vaccinated mouse/SFU for the same peptide in naïve mouse. The SI ≥ 2 considered as a positive response.
